# Whole genome sequence analysis of shallot virus X from India reveals it to be a natural recombinant with positive selection pressure

**DOI:** 10.1186/s12863-024-01196-z

**Published:** 2024-05-06

**Authors:** Jyoti Singh, Sachin Teotia, Ajay Kumar Singh, Meenakshi Arya, Ajaya Kumar Rout, Bijay Kumar Behera, Shahana Majumder

**Affiliations:** 1 Department of Biotechnology, Sharda University, Greater Noida, India; 2 Department of Botany, School of Life Sciences, Mahatma Gandhi Central University, Motihari, Bihar India; 3 Deaprtment of Bioinformatics, Central University of South Bihar, Gaya, Bihar India; 4 Rani Lakshmi Bai Central Agricultural University, 284003 Jhansi, Uttar Pradesh India

**Keywords:** Shallot virus X, Next generation sequencing, Recombination, Positive selection

## Abstract

**Background:**

Shallots are infected by various viruses like Onion yellow dwarf virus (OYDV), Leek yellow stripe virus (LYSV), Shallot latent virus (SLV) and Shallot virus X (ShVX). In India, they have been found to be persistently infected by ShVX. ShVX also infects onion and garlic in combination with other carlaviruses and potyviruses. ShVX is a member of genus *Allexivirus* of family Alphaflexiviridae. ShVX has a monopartite genome, which is represented by positive sense single-stranded RNA. Globally, only six complete and 3 nearly complete genome sequences of ShV X are reported to date. This number is insufficient to measure a taxon’s true molecular diversity. Moreover, the complete genome sequence of ShVX from Asia has not been reported as yet. Therefore, this study was undertaken to generate a complete genome sequence of ShVX from India.

**Results:**

Shallot virus X (ShVX) is one of the significant threats to Allium crop production. In this study, we report the first complete genome sequence of the ShVX from India through Next-generation sequencing (NGS). The complete genome of the ShVX (Accession No. OK104171), from this study comprised 8911 nucleotides. *In-silico* analysis of the sequence revealed variability between this isolate and isolates from other countries. The dissimilarities are spread all over the genome specifically some non-coding intergenic regions. Statistical analysis of individual genes for site-specific selection indicates a positive selection in NABP region. The presence of a recombination event was detected in coat protein region. The sequence similarity percentage and phylogenetic analysis indicate ShVX Indian isolate is a distinctly different isolate. Recombination and site-specific selection may have a function in the evolution of this isolate. This is the first detailed study of the ShVX complete genome sequence from Southeast Asia.

**Conclusion:**

This study presents the first report of the entire genome sequence of an Indian isolate of ShVX along with an in-depth exploration of its evolutionary traits. The findings highlight the Indian variant as a naturally occurring recombinant, emphasizing the substantial role of recombination in the evolution of this viral species. This insight into the molecular diversity of strains within a specific geographical region holds immense significance for comprehending and forecasting potential epidemics. Consequently, the insights garnered from this research hold practical value for shaping ShVX management strategies and providing a foundation for forthcoming studies delving into its evolutionary trajectory.

## Introduction

Shallot (*Allium cepa* var. *aggregatum* G. Don), also known as small onion/multiplier onion, is a culinary herb. It is one of the most important spice crops in the world and is used for seasoning dishes. It is a member of the Alliaceae family and is closely related to onion, garlic and chives. It is mainly grown in China, Southeast Asia, and Europe [1]. The multiplier onion is considered more nutritive than a bigger variety in terms of carbohydrates, protein, carotene and minerals [2]. Several studies have indicated that shallots have higher quantities of polyphenols, flavonoids and other phytochemical substances linked with antioxidant and anti-fungal activity than other *Allium cepa* L members [3].

Deterioration in quality and losses in crop yields due to viral infection are common problems encountered by growers [4]. Shallots are infected by various viruses like Onion yellow dwarf virus (OYDV), Leek yellow stripe virus (LYSV), Shallot latent virus (SLV) and Shallot virus X (ShVX). In India, they have been found to be persistently infected by ShVX [5]. It also infects onion and garlic in combination with other carlaviruses and potyviruses [6–8]. By jumping host back and forth, the virus may modify itself to accommodate various plant systems and the presence of multiple viruses simultaneously may catalyze intra- and inter-species recombination. Therefore, this study was undertaken to record the molecular structure of an Indian isolate of this less explored virus.

**Fig. 1:**
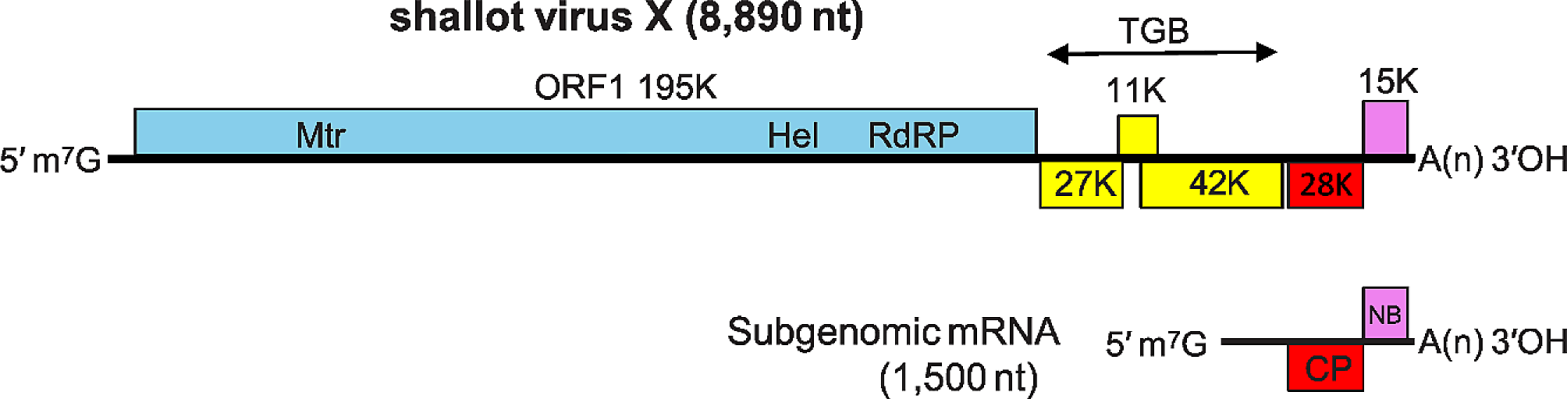
Schematic representation of ShVX genome (https://ictv.global/report/chapter/alphaflexiviridae/alphaflexiviridae/allexivirus)

ShVX, is the member of genus *Allexivirus* of family Alphaflexiviridae [9]. No distinct symptoms are known to be induced by ShVX and are transmitted by the dry bulb mite (*Aceria tulipae*) [6]. ShVX has a monopartite genome, which is represented by positive sense single-stranded RNA [10]. The ShVX virions are highly flexible filamentous particles with an 8890 nucleotide genome, measuring around 800 nm in length and 12 nm in diameter [11, 12]. The genome has six Open Reading Frames (ORFs), ORF1 codes for 170–195 kDa alphalike replicase (consist of helicase, RNA-dependent RNA polymerase and methyl transferase). ORF2 and ORF3, code for 26 and 11 kDa proteins respectively and helps in cell to cell movement [11]. These are identical to the triple gene block proteins TGB1 and TGB2 discovered in a variety of plant viruses [13]. ORF4 encodes P42, a 42 kDa serine-rich protein with unclear function and no orthologous genes discovered in other allexiviruses or closely related genera such as Potexvirus or Carlavirus [14]. ORF5 codes for the coat protein, which is 28 kDa in size, while ORF6 encodes 15 kDa nucleic-acid binding protein [11] (Fig. 1).

ShVX has been found infecting shallot in various countries like Netherlands, New Zealand, Sudan, Italy, India and Poland [6, 7, 15–18]. But globally, only six complete and 3 nearly complete genome sequences of ShVX are reported to date which are from USA, Russia, France and Slovenia. This number is insufficient to measure a taxon’s true molecular diversity. Moreover, complete genome sequence of ShVX from Asia has not been reported as yet. Therefore, this study was undertaken to get whole genome sequence of ShVX infecting shallots in India and to investigate its molecular structure to understand its distinctness, trace its phylogeny and add to the existing knowledge so as to help in epidemiological studies.

## Materials and methods

### Field sample collection

Surveys of shallot growing fields were done to collect shallot samples with the aim to detect and identify *Allexivirus* infection in shallot. A total of 15 samples were collected from various fields of Kerala and Tamil Nadu in 2019 and were brought to Sharda University, Greater Noida, U.P. Leaf samples from each plant were transported to lab on dry ice and stored at -20℃ (Table 1). The bulbs were transported at room temperature and stored in insect free chamber for further use. Shallot plants raised from seed and grown in insect free glass house was used as healthy sample.

### Detection of an *Allexivirus*

The presence of allexiviruses were detected by RT-PCR using group specific primers published by Dovas et al. 2001. Total RNA was extracted from 100 mg of tissue from a shallot bulb using RNeasy Plant Minikit (Qiagen) according to the manufacturer’s protocol. The quality of RNA (using 260/280 ratio) and concentration was determined using UV visible double beam spectrophotometer (Shimadzu-1800, Japan). The first strand of cDNA was prepared using RevertAid Reverse Transcriptase (Thermo Fisher Scientific) as published earlier [15]. RT-PCR was performed using 10 µl of RT reaction mixture, 1 µl of Taq DNA polymerase (Thermo Fisher Scientific), 5 µl of 10X reaction buffer, 3 µl MgCl_2_, 2 µl primer pair (forward and reverse) and 1 µl dNTPs. The temperature profile consisted of a denaturation step at 94 °C for 5 min, then 30 cycles of 45 s at 94 °C, 20 s at 60 °C and 1 min at 72 °C and one final extension step at 72 °C for 10 min. Ten microlitres of amplified product were separated by electrophoresis in a 1% agarose gel containing ethidium bromide and photographed under UV illumination with an imaging system (BioRad Gel Doc EZ Imager). The expected amplicon of ~ 200 bp fragment was obtained. PCR products were sent for sequencing to Barcode Biosciences, Bangalore, India.

### RNA extraction, library preparation and Illumina HiSeq

Total RNA was isolated, as described earlier from sample no. 14 collected from Punalur district of Kerala, India (Table 1). The RNA was eluted in 30 µl of RNase free water. RNA was sent for sequencing using Illumina HiSeq 2000 platform, to Barcode Biosciences, Bangalore. The quality of RNA was checked by using The Agilent 4150 TapeStation system and the quantity of RNA was checked by using The Qubit® Fluorometer. Depending on the peaks, the integrity of RNA was determined by RNA integrity number (RIN). RIN values were measured from 1 to 10, where RIN Value of 1–5 indicates complete degradation 5–7 indicates partially degraded RNA and RIN value above 8 indicates good quality RNA. Ribosomal RNA was depleted from 1uG of total RNA using a Ribominus plant kit for RNA seq (Thermo Fisher Scientific) and total RNA libraries were generated using the manufacturer’s instructions for an Illumina NEBNext® UltraTM II RNA Library Prep Kit at Barcode Biosciences, Bangalore.

### Data processing

The base call files were converted to fastq files using bcl2fastq program. The raw data in FASTQ format was subjected to data QC by FastQC and MultiQC following the adapter and low-quality trimming by using fastp. The trimmed reads are analyzed using KRAKEN2 51 for taxonomical classification using the viral reads (RefSeq) available from the NCBI nucleotide database [19]. The reads were assembled into contigs using IDBA-UD [20]. IDBA-MT was used to remove the chimeric contigs [21]. The final contigs generated by IDBA-MT were searched for protein homology against uniref100 protein database using Diamond [22] BLASTx. The contigs that showed homology with the protein of interest were extracted and clustered using CD-HIT-EST with default parameters. The obtained contigs were annotated using Prokka v 1.3. The annotated protein sequences were searched for homology against Uniref100 to obtain their functional information using BLASTp algorithm of Diamond program by denoting the kingdom option as Viruses. Contigs were assembled using Bio-Edit 7.0 program and a complete genome sequence of ShVX was constructed.

### Validation of sequencing result

To validate ShVX infection in shallot plants, PCR primers targeting the Replicase, TGB1, CP and NABP regions were designed using the whole genome sequence of ShVX obtained in this study (Table 2). RNA was isolated and RT-PCR reaction was performed as described in above section. The PCR amplicons of expected size were sequenced at Barcode Biosciences. Each sequence was identified using BLASTn (Nucleotide BLAST) and was also compared with the whole genome sequence of ShVX obtained in this study.

### Sequence similarity and phylogenetic analysis

Initially, BLASTn programme was used for preliminary identification. The nucleotide and amino acid sequence percent similarities were calculated by using Bio-Edit 7.0 program. A pairwise sequence identity percentage graph of the isolate under study with other allexiviruses was prepared by using Sequence demarcation tool (SDTv1.2).

The phylogenetic tree analyses were performed using ShVX sequence obtained from this study along with complete genome sequences of ShVX isolates retrieved from NCBI. The ClustalW in MEGA 11 software was used to align the sequences and the trees were generated using the NJ method using Jukes-Cantor model of evolution. The statistical significance of each node was determined with 1000 bootstrap replicates.

### Evolutionary data analysis

Open reading frames (ORFs) were predicted using ORF finder (https://www.ncbi.nlm.nih.gov/orffinder/). The conserved domains in the predicted proteins were investigated using motif finder (https://www.genome.jp/tools/motif/). To better understand the impact of selection pressure on each ShVX gene, the ratio of nonsynonymous (Ka) to synonymous substitution (Ks) was calculated using Ka/Ks calculator [23]. To test for positive selection, *d*N and *d*S values for triplet codon of sequences were calculated using the random effect likelihood method available in HYPHY [24], implemented in MEGA 11 software. For recombination analysis, the Recombination Detection Program v.4.16 (RDP4) was employed. The RDP, GENECONV, Chimaera, MaxChi, BOOTSCAN, PhylPro, LARD and SISCAN methods implemented in the RDP4 programme with default parameters were used to assess recombination events, likely parental isolates of recombinants, and recombination break points.

## Results

### Detection of an *Allexivirus*

The RT-PCR amplification of the section of coat protein gene and NABP gene using previously published group specific primers revealed amplicons of ~ 200 bp in all the 15 samples collected from Kerala and Tamil Nadu. No amplification was obtained from healthy plants. BLASTn analysis of sequences obtained from PCR products revealed that the 10 shallot samples from Tamil Nadu and 5 shallot samples from Kerala were infected with allexiviruses. Sequences from three samples from Tamil Nadu (5, 7, 8) and two samples from Kerala (13,14) were found to be more than 90% similar to ShVX (Table 1).

**Table 1:** Allexiviruses present in accessions collected from different states of India

S.No	Region	State	Accessions	Allexiviruses	% Similarity with isolates available in NCBI
1	Dindigul	Tamil Nadu	Agrifound red	+	88.7–93.8
2	Dindigul	Tamil Nadu	CO1	+	88.9–92.3
3	Dindigul	Tamil Nadu	CO1	+	89.3–92.9
4	Dindigul	Tamil Nadu	COOn5	+	87.8–92.2
5	Dindigul	Tamil Nadu	COOn5	+	89.3–92.6
6	Trichy	Tamil Nadu	CO4	+	88.6–94.3
7	Trichy	Tamil Nadu	CO4	+	87.9–93.4
8	Trichy	Tamil Nadu	CO5	+	89.0-94.2
9	Trichy	Tamil Nadu	CO6	+	87.6–93.3
10	Trichy	Tamil Nadu	CO6	+	87.8–93.6
11	Punalur	Kerala	Agrifound red	+	91.1–96.3
12	Punalur	Kerala	Agrifound red	+	91.6–96.6
13	Punalur	Kerala	Agrifound red	+	93.3–97.6
14	Punalur	Kerala	Agrifound red	+	93.8–98.7
15	Punalur	Kerala	Agrifound red	+	93.5–98.6

### Illumina HiSeq and virus analysis

The RNA provided had a RIN value of 7.5, which is optimal for library preparation. After trimming, 63,544,172 reads (with an average length of 150 bp) were obtained from the roughly 65 million 150 bp paired-end reads generated by Illumina sequencing in two libraries. The 11 contigs that showed homology with ShVX were clustered into 8 contigs by using CD-HIT-EST. The total length of the contigs was 20,296 with minimum and maximum contig sizes of 285 and 10,719 respectively. A total of 17 proteins were annotated and were assigned as hypothetical proteins, replicase, 40 kDa protein, movement and silencing protein and coat protein. The obtained sequences were assembled and the overlapping fragments were collapsed to obtain a complete genome sequence of ShVX (excluding poly A tail), by using Bio-Edit 7.0 program. Through Illumina, a 7901 nt long, a single contig was obtained for ShVX genome. The complete genome of the ShVX isolates from this study was constructed by overlapping this contig and other smaller contigs using Bio-Edit 7.0 program. A consensus full genome sequence for the isolate from this study was found to comprise 8911 nucleotides.

The genome sequence after annotation was predicted to contain 5′UTR (1-104 nt), the RNA-dependent RNA Polymerase(105-5270nt), Triple gene block 1 (TGB1) protein (5335-6060nt), TGB2 protein (6038-6349nt), Serine rich (P42) TGB3 protein(6460-7602nt), coat protein (CP)(7626-8414nt), Nucleic acid binding protein (NABP) (8415-8800nt) and 3′UTR (8801-8911nt).

### Validation of sequencing result

Amplicons from validation PCR were of expected sizes and when directly sequenced showed similarity with targeted genes (Table 2). Thus, verified complete genome sequence obtained in this study was submitted to GenBank under accession no. OK104171.

**Table 2:** The primers used to amplify different ORFs of ShVX. The size of the PCR product and the similarity of the obtained sequence with the respective isolates present in NCBI and with the whole genome sequence obtained in this study

S. No	Targeted gene	Primer pairs with nucleotide position	Amplified product size	% similarity using BLASTn	% similarity with WGS from this study
1.	Replicase	Forward (130–146) 5^’^-GACCAAATTAGCGACCC-3^’^ Reverse (1219–1235) 5^’^-CCGAATAAATTCTCGAG-3^’^	1.1 kb	80.74–81.7	100
2.	TGB-1	Forward (5234–5248) 5^’^-AACATCTTGGGAAGG-3^’^ Reverse (6225–6240) 5^’^-GTTGAAGCTCCAGTT-3^’^	~ 1Kb	77.4–79.5	98.9
3.	Coat protein	Forward (7574–7588) 5^’^-TTTACAAATTTAGGG-3^’^ Reverse (8476–8492) 5^’^-AGTTTTGAGGTCGTTGG-3^’^	918 bp	92- 87.5	100
4.	NABP	Forward (8347–8361) 5^’^-TCAACTAGAGCCGAC-3^’^ Reverse (8871–8890) 5^’^-GAGTAAGTTTAGCTATATCA-3^’^	543 bp	88-95.2	99.8

### Sequence similarity and phylogenetic analysis

**Fig. 2:**
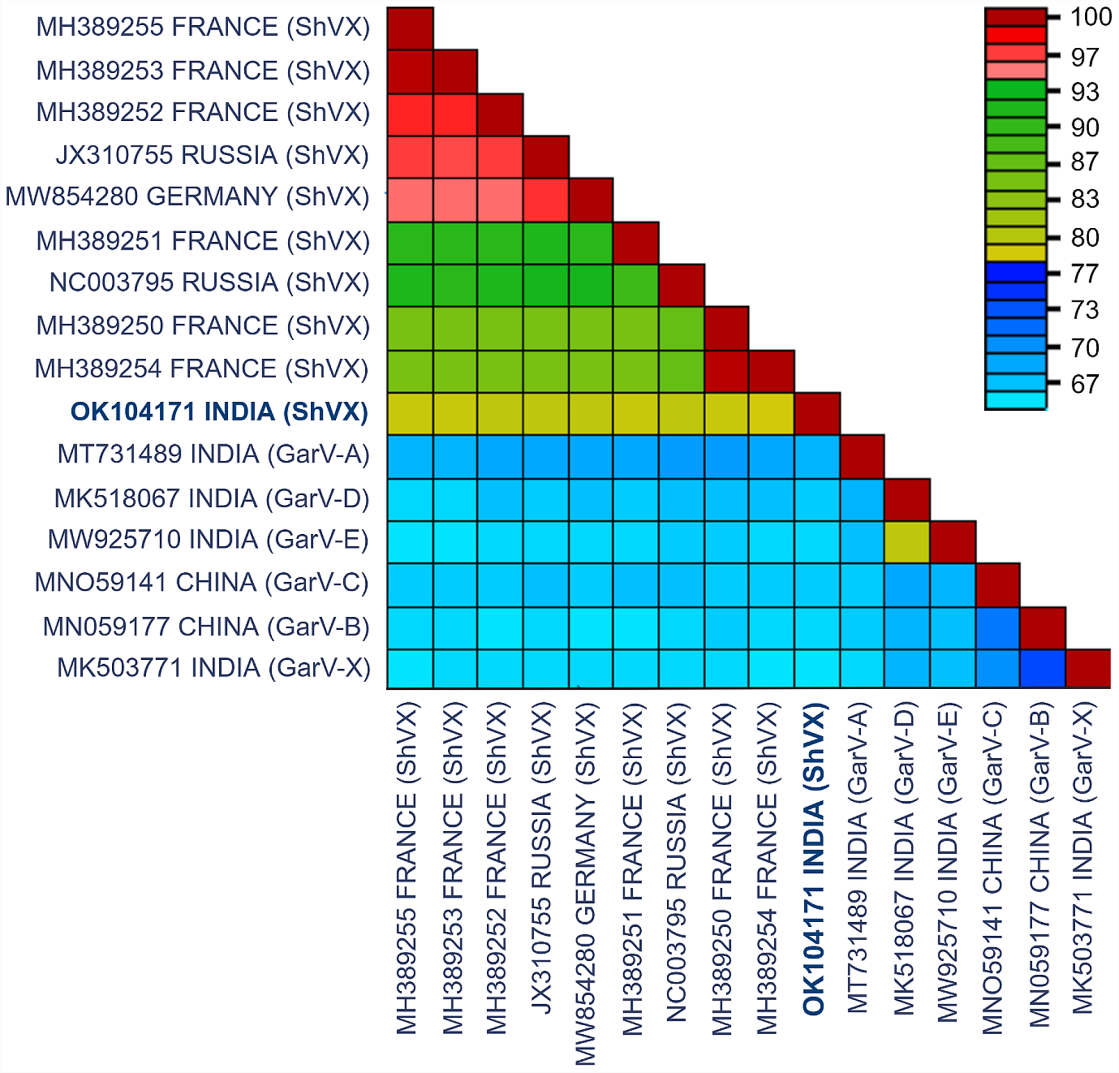
Pairwise identity plot of complete genome sequences of 16 allexiviruses including one from this study (OK104171), aligned by ClustalW and visualised with Sequence Demarcation Tool software. The colours on the scale represent the pairwise identities on the coloured heat map

**Fig. 3:**
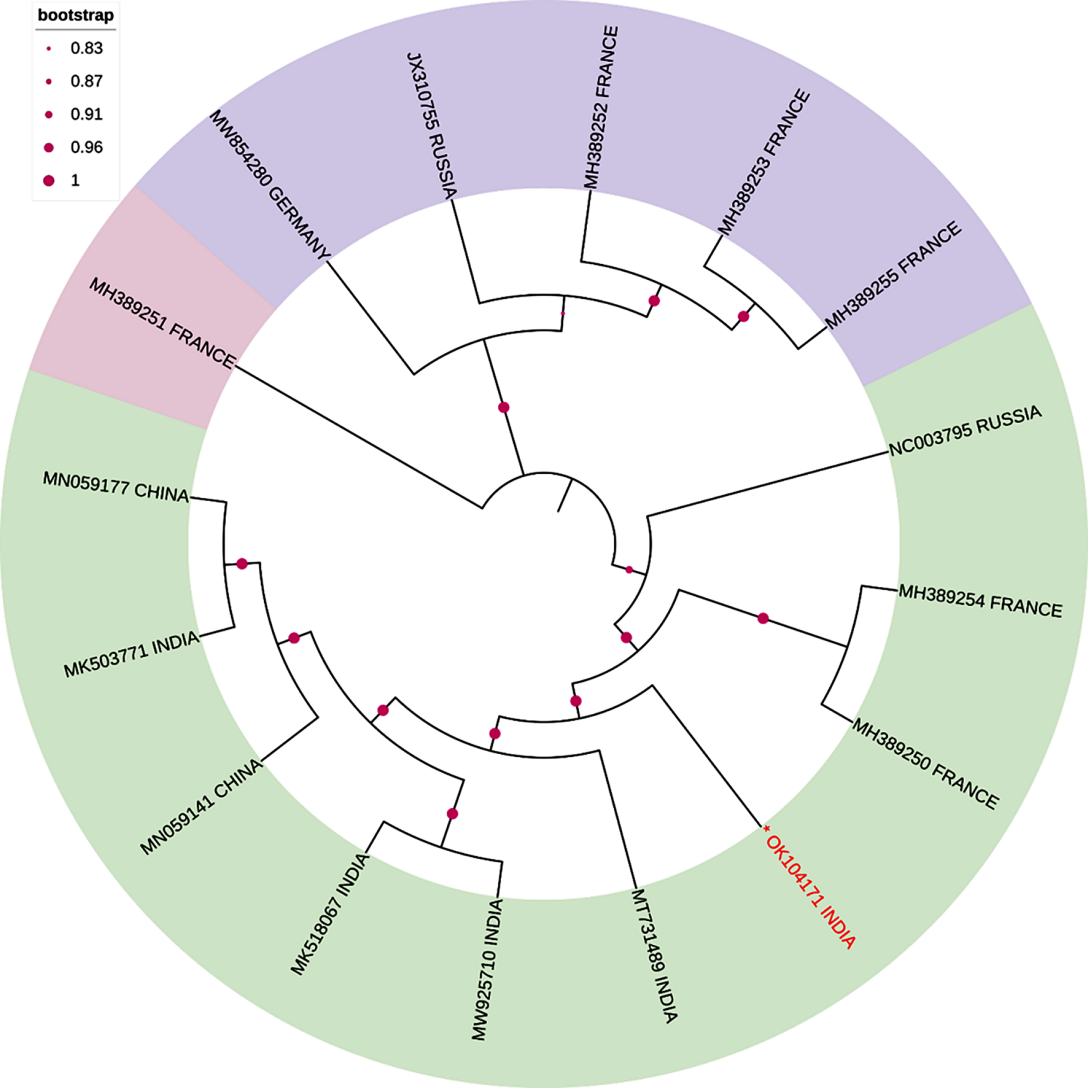
The Neighbor-Joining approach was used to infer the evolutionary history. The evolutionary history of the taxa investigated is represented by the bootstrap consensus tree, which is derived from 1000 replicates. The Maximum Composite Likelihood approach was utilized to calculate the evolutionary distances, which are expressed in the units of base substitutions per site. The present study includes a total of 16 nucleotide sequences. The final dataset consisted of a total of 9243 locations. Evolutionary analyses were performed using the MEGA 11 software

Blast analysis revealed that the complete genomic sequence of ShVX shared 79.37–81.17% sequence identities with whole genome sequences of other ShVX isolates. Pairwise sequence identities (%) of ShVX (OK104171) complete genomic sequence shared 75-78.9% nucleotide (nt) identities with whole genome sequences of other ShVX isolates reported globally. The isolate under study shared the highest pairwise sequence identity at a nucleotide level of 79.2% with an isolate from Russia (JX310755) while the lowest pairwise sequence identity of 78.1% was found with an isolate from France (MH389251). The BLASTn analysis of individual ORFs indicated that they are homologous to ORFs of other ShVX isolates present in NCBI. The highest similarity is shown by NABP gene with more than 94% similarity with NABP gene of other ShVX isolates. The least similarity of 71.3–73.8% was observed in P42 gene. When the CP of the isolate was compared to previous ShVX isolates available in GenBank, sequence identity values (80.4 to 92.1% nt and 84 to 95.6% aa) were higher than the thresholds defined by the ICTV (minimum of 72% of nt identity or 80% of aa identity of the CP or the polymerase gene) for species demarcation, hence it is considered an isolate of ShVX. Complete genome sequence of 26 allexiviruses including one from this study (OK104171) was used for sequence diversity analysis. The graph generated from SDTv1.2 showed that our isolate shared around 78–79% identity with ShVX isolates from around the world and it shared less than 70% identity with other allexiviruses (Fig. 2). Phylogenetic tree of whole genome sequence and all the ORFs (data not shown) showed that our Indian isolate does not group with any other isolate from around the world rather forms a separate branch. The phylogenetic tree also revealed that clustering among ShVX isolates was independent of geographical area. When compared to other allexiviruses, GarV-A, was found to be a little more similar to our isolate than the GarV-B, GarV-C, GarVD, GarVE and GarV-X sequences. The phylogenetic tree was constructed using the MEGA 11 software [25] (Fig. 3). It is also supported by the results obtained through SDTv1.2 (Fig. 2).

### Evolutionary data analysis

Each ORF encoded by the ShVX genome possess Allexivirus-specific conserved motifs which are described in literature (Table 3). Based on the tree generated by the *K*a/*K*s calculator, using dataset prepared from representative nucleotide sequences of each ORF, selection pressure for ORF1-5 was found to be negative (ka/ks < 1) (figure not shown), whereas ORF 6 showed positive selection pressure (ka/ks > 1) with 1.7701 ka/ks value. One triplet codon, ACT (nt73-75) with p-value of 0.45 was identified in ORF6 which is undergoing positive selection.

The analysis of whole genome sequence using RDP4 software revealed the presence of one possible recombination event. The presence of recombination event was detected with seven of nine methods implemented in RDP4, with p-value less than 0.05. The RDP4 program detected two isolates, MH389250 and MH389250 from France as major and minor parental sequences respectively, of the isolate under this study. It showed 94.9% level of similarity with minor parent. Positions 7679 and 8406 were identified as the start and end of recombination breakpoints during the investigation which resides in the coat protein region. The recombination events were also detected in whole genome sequences of other ShVX isolates used for analysis (Table 4).

**Table 3:** Characterizations of six ORFs of ShVX isolate under study

S.No	ORF	Length (aa)	Components	Conserved motif Sequence	Indicative of
1.	ORF1	1721	Methyltransferase (40–336)	L-X6-LX6-L-X6-L	Methyl transferase
			Helicase (914–1145)	GxxGxGKS	Viral helicase
			RdRp (1267–1600)	SGX3TX3NTX18-37GDD	RNA-dependent RNA polymerase
2.	ORF2	241	Triple gene block 1 (TGB-1)	GVPGSGKS	RNA helicase
3.	ORF3	103	Triple gene block 2 (TGB-2)	Movement protein domain	Cell to cell movement
4.	ORF4	380	P42		Predicted to be involved in intracellular mobility
5.	ORF5	262	CP	Flexi_CP	Coat protein
6.	ORF6	137	Nucleic acid binding protein (NABP)	CFDCGAYLYDDHVCKRFTSRSNSDCLSVIH	Zinc finger motif.
				CCxH	Cysteine rich protein (CRP)

**Table 4:** Summary of recombination analysis of whole genome sequence of ShVX Indian isolate along with homologous sequences retrieved from NCBI using RDP4.

			Breakpoints		Detection	
Recombinant	Major parent	Minor parent	Begin	End	method	p-value
OK104171	MH389250	MH389252	7679	8406	RDP	2.417*10^− 19^
India	France	France			GENECONV BootScan MaxiChi Chimaera SiScan 3seq	2.229*10^− 17^ 3.320*10^− 26^ 8.887*10^− 12^ 3.047*10^− 11^ 4.709*10^− 17^ 3.996*10^− 14^

## Discussion

Complete genome sequencing is an important tool to understand the molecular characteristics of an organism and its dynamics. It allows researchers to address fundamental biological questions, predict novel putative genes, predict and prove functions of genes, easily clone and express any gene, understand virulence determinants and molecular adaptations to various external factors [26]. Since 2009, next-generation sequencing (NGS) has been widely used in plant virus research [27]. It is frequently used for uncovering full novel viral sequences and obtaining complete genome sequences for known viruses [28]. NGS is a user-friendly and more efficient technology with excellent resolution as compared to conventional Sanger sequencing for obtaining whole virus genomes in terms of cost and time requirements [29].

Shallots are propagated asexually using bulbs, therefore once infected by any virus acts as a storehouse for that virus in next generation. Shallots are known to be infected by at-least two viruses [30]. Infection of a host with multiple viruses simultaneously makes them suitable workshops for intra- and inter-species recombination. This may always lead to emergence of new strains with altered characters [31, 32]. Constant monitoring of a viral strain is essential to prevent an epidemic. Therefore, to keep track of the changes we need to have complete information on its molecular structure.

Therefore, in this study, we used NGS technique to obtain the first complete genome of ShVX from Indian subcontinent and Southeast Asia. The whole genome sequence of ShVX isolates investigated in this study is only 80% similar to the other six whole genome sequences available in GeneBank. Major dissimilarities were found in the region of Replicase gene and P42 gene. These dissimilarities were detected in comparison with only nine other ShVX sequences and so we are unable to predict any trend. Though these stretches of sequences in both genes lies in the non-coding region, therefore, may not have any immediate effect on the host pathogen interactions but their functional role should not be rejected [33, 34]. However, these changes if could be found in a larger number of Asian samples may indicate some evolutionary progress. The sequence similarity analysis and phylogenetic trees based on the complete genome sequence indicate that ShVX from India is a distinctly different isolate. Individual phylogenetic tree for each gene of the ShVX isolate revealed a tree topology that was similar to that obtained from complete genome sequences and thus supports the distinct isolate claim.

This study indicates the isolate to be a recombinant at coat protein region. Selection pressure analysis though performed with a small dataset indicates a positive or directional selection pressure on ORF6 (NABP region). The positive selection of residue ACT (nt73-75) indicates that the residue is under a diversifying selection and might be relevant for its evolution. All this information indicates that the isolate under study is undergoing diversification. Due to the lack of other sequences from Southeast Asia or India it is difficult for us to predict its evolutionary path or its origin but it will provide critical information for predicting mutational patterns to understand the direction of evolution in future research.

To control viral diseases, it is necessary we understand the dynamic molecular architecture of their genome and keep a track of their evolution. A genome-based constant monitoring system for viruses is essential to predict and prevent any epidemic [35–37]. Since only six complete genome and 3 nearly complete genome sequences are available, the whole genome sequence information of this distinct Indian ShVX will contribute in that direction. This knowledge will be important for future molecular epidemiological studies.

## Conclusions

In conclusion, we want to emphasise that this is the first report of complete genome sequence of an Indian ShVX and its detailed evolutionary characterisation. This study indicates that the Indian isolate is a natural recombinant and so recombination is a major evolutionary force for this virus species. Information regarding molecular diversity of isolates from a geographical area is often crucial to understand and predict epidemic. Therefore, information generated in this study will be useful in ShVX management strategy, as well as future evolutionary investigations of ShVX.

## Data Availability

The datasets generated during the current study are available in the GenBank repository, [https://www.ncbi.nlm.nih.gov/nuccore/OK104171.1]
